# The Ultimate Results of Inoperable Gynæcological Cases

**Published:** 1907-12-21

**Authors:** A. E. Giles

**Affiliations:** Surgeon to the Chelsea Hospital for Women.


### Hospital Clinics.

### THE ULTIMATE RESULTS OF INOPERABLE GYNÆCOLOGICAL CASES.

By A. E. GILES, B.Sc., M.D., F.R.C.S., Surgeon to the Chelsea Hospital for Women.

Abstract of a Lecture delivered at the North East London Post-Graduate College, November 28, 1907.

In speaking to-day of inoperable cases I shall include a certain number which are not, strictly speaking, gynæcological cases at all. One reason for this is that before opening the abdomen it is sometimes impossible to say whether a given tumour is connected with the pelvis or not; and another is that in disease disseminated both in the abdomen and the pelvis it is not always easy to say which is the primary focus. I. shall include, therefore, all those cases in which I have found at operation an irremovable tumour, and have been obliged to leave it quite alone.

The points which guide one as to the diagnosis and prognosis of any given tumour before laparotomy are as follows: If the patient is not over forty, if she is not losing flesh, if there is no free fluid in the peritoneum, and if the tumour is felt to be a single one, these are the points which are favourable. Unfavourable signs in prognosis are the reverse; multiplicity, wasting, free fluid in the peritoneum, and an age greater than forty.

If, then, in any given case there is a probability of malignancy, we are confronted with the question whether to operate or not. Unless the patient is *in extremis*, I recommend operation for many reasons. (1) Diagnosis is frequently fallacious, and even after long experience it is possible often to make mistakes. To leave, unoperated upon, a woman with a removable benign tumour is a grave mistake; for instance, a simple ovarian cyst will prove fatal if nothing is done. Moreover, it has repeatedly happened to me to operate, expecting to find a malignant growth, and then to discover it to be benign. (2) Even if the case turns out inoperable, one may be able to do some good; thus the mere removal of hydroperitoneum is often a great gain to the patient. You may say the same end can be achieved by tapping; but tapping is not so valuable, partly because you cannot make certain of the diagnosis that way, partly because for some unknown reason the fluid takes a much longer time to reaccumulate after an open operation. (3) There are a few extraordinary cases in which after laparotomy the patient apparently loses her tumour; I shall refer to instances of this presently. This is not an event one can count on, but it is a point worth bearing in mind when there is any doubt.

Having got as far as opening the abdomen, we are often at a loss to determine whether a tumour is malignant or innocent. There is one type of tumour—papilloma of the ovary—which in its physical characteristics looks distinctly malignant in respect of secondary growths, ascites, etc., and yet clinically is often quite innocent. If the tumour is a single one, there is always a good chance for the patient; for even though it be actually malignant, if it is sufficiently circumscribed to be removed entire there is a good prospect of cure. But if the growth is multiple, the outlook is very different. A growth, for example, affecting the ovaries, spreading to the uterus, with similar growths in the mesentery, glands, stomach, etc., may be at once said to be malignant, and one for which nothing can be done. This leads me to the question of what justifies one in closing the abdomen without removing a tumour. One has, of course, to ignore the personal element, for one surgeon may be appalled by difficulties which would not deter another. I have never to my knowledge "funked" an innocent growth, but I have often left malignant ones alone. If the growth involves, say, a large section of the liver, or affects important organs and is multiple, or there seems no chance of removing the whole growth, those are the indications for doing nothing. One more point. Malignant tumours are often very vascular, and if you begin to interfere you may set up most severe hæmorrhage; this does not matter if you see your way clear to complete the operation, but it may happen that the surgeon is confronted with the impossibility of doing so, and with uncontrollable hæmorrhage too.

Now I come to the main point. What happens to these patients afterwards? I have deliberately left tumours untouched in twenty-three cases. Two of these are not quite in the same category as the others. One was a case of fibroids where, after removing the uterus, there was revealed a growth of the bowel which, considering the severe operation already performed, I did not feel justified in removing. The other case was also one of two distinct growths.

Of the remaining twenty-one cases, seven I have not been able to trace. Of the fourteen left, two are alive and well at the present time, one is alive but losing ground, and the remaining eleven have died at varying intervals. These intervals have been in one case three and in another four days. These are virtually operative deaths, though both patients were in an advanced stage of disease, and the operation did not very greatly hasten the end. One death occurred at nine days, others at three weeks, seven weeks, two and a half months, three months, and the rest up to eleven months.

An important question is how far these patients were improved. I know the recital of cases is tedious, and I shall therefore run very briefly over some of them which are interesting. In August 1895 I operated on a case which was diagnosed clinically as one of double malignant disease, and found ovarian papillomata with hydroperitoneum; the growths were extensively distributed. This patient died four months afterwards, and her doctor reported that she was tapped two or three times before her death.

Another patient who had an abdominal tumour was in addition four and a half months pregnant. When operated on in January 1902 an apparently malignant mass was found occupying the omentum, with extensions to the colon, stomach, and liver. She got gradually weaker, aborted on the twenty-first day, and died on the twenty-second.

A third case was that of a woman aged fifty, who had extensive papillomata of the ovaries and abdominal cavity with hydroperitoneum. The abdomen was drained, but she died nine days after. Her doctor reported that she was very considerably relieved by the operation, and even though it was only for a few days, at least that is something.

A woman with a double abdominal tumour was, in January 1904, submitted to laparotomy, and the condition found was taken for a malignant mass in connection with each kidney. Much to my surprise, when making inquiries for the purposes of this lecture, I got a reply from her in July 1907. I was still further astonished to hear that she had a baby in 1906. On examining her I could find nothing abnormal on the left side, and on the right a mass about twice as large as the normal kidney. I fancy this must have been an instance of double congenital cystic disease of the kidneys.

In July 1904 I operated on a case of double ovarian carcinoma; the patient died nine months afterwards.

In January 1905 a woman aged thirty came to the Chelsea Hospital for Women. On opening her abdomen I found abundant hydroperitoneum and extensive papillomata, with deposits all over the abdomen on both sides. After sponging out the fluid I shut up the abdomen without removing any of the growths. This patient also turned up this year in reply to a letter of inquiry, and her history was even more interesting than that of the other woman. She said she felt extremely well, and she had gained three stone in weight. Some time after I had operated on her, as she was doing badly, her employer suggested to her that she should see Mr. Douglas Harmer, of St. Bartholomew's Hospital. He admitted her to the hospital, and removed something, after which she got quite well. I wrote to Mr. Harmer about the case, and I will read portions of his letter of July 1907:—

"She was very ill when she came"—that is in June 1906, a year and a half after my operation—"and seemed from her history to be going down. I advised exploration, as it seemed rather a long history for malignant disease. There was much fluid in the abdomen, and a papillomatous growth on the right side of the pelvis. The whole mass was removed and also a small uterine fibroid. Pathologically it was reported to be a cyst of the ovary lined by squamous epithelium."

It is difficult to compare the state of the patient at the time of the second operation with that at the time of the first, but we may notice that Mr. Harmer found the disease affecting the right side, whereas previously it was all over. I think I may say that my only feelings about this case were those of satisfaction that he succeeded where I failed.

The next case was that of a woman who had been repeatedly tapped to the extent of over 150 pints. At operation in July 1905 there were numerous carcinomatous growths in the loin and pelvis, and it was evident that nothing could be done. The fluid reaccumulated much more slowly, and several months elapsed before she again required tapping, whereas previously this had been necessary every fortnight. She died in January 1906.

Then I had a case of inoperable carcinoma, which died five months after operation. After that I saw a woman of forty-six, one of the two whom I excluded at the commencement; she had a retroperitoneal growth, and also one of the duodenum. She died seven weeks after her laparotomy.

In November 1906 there was a case of extensive retroperitoneal tumour, with much hydroperitoneum, a combination which is unusual. The chief interest about her is the length of time after operation before she required tapping. This was in February 1907; after that she was tapped oftener, and died in October of this year.

A very peculiar case was another retroperitoneal growth involving the illocæcal junction, and suppurating acutely. This patient survived operation for two and a half months.

Now I come to the case of a single lady aged fifty-two. She was operated upon in June 1907, having been explored in the North of England about two months previously. She was very anxious that something more should be attempted, but there turned out to be extensive malignant disease all over the pelvis. Although the patient had not apparently improved after the first operation, she did so after the second, and I tapped her for the first time only two days ago, five months subsequent to the operation. Whether this long interval means a better prognosis one cannot say, but it seems possible.

Yet another patient was thirty-three years old only. Note this as possibly bearing on the result. She had a tumour diagnosed as ovarian: it turned out that it consisted of two parts, one a tubo-ovarian cyst, which was removed; the other a tumour, apparently malignant, in the mesentery, which was left. The operation was in January 1903. In July 1906 the last time I saw her, she was apparently well, and had no sign of any tumour.

The last case was one of fibroids admitted to this hospital in May 1903. Hysterectomy was done, and after concluding this operation I found a tumour of the sigmoid about the size of a large hen's egg, having all the appearances of a carcinoma. I decided to close the abdomen and await developments. I saw the patient in July 1907, and up to then she had had neither symptom nor sign of any intestinal trouble; not even more constipation than falls to the lot of the average woman.

Now what are we to make of these tumours that disappeared? We must always remember that the diagnosis is not, as a rule, verified microscopically. It is easy to say that recovery proves the tumour to have been a simple one, and death proves it to have been malignant; but the reasoning is fallacious. For some simple tumours, if left alone, will prove fatal, and this is not proof that such tumours are malignant; while you may have come across in your reading other cases in which patients suffering from undoubted malignant disease have got quite well without treatment. We must be content with the assumption that such cases were malignant, but that for some obscure reason they have got well. The outcome of it all is, in my opinion, that exploration in this kind of case is always worth while unless the condition of the patient forbids it.

